# *In Vivo* Near-Infrared Fluorescence Imaging of Atherosclerosis Using Local Delivery of Novel Targeted Molecular Probes

**DOI:** 10.1038/s41598-019-38970-4

**Published:** 2019-02-25

**Authors:** Marie-Jeanne Bertrand, Maxime Abran, Foued Maafi, David Busseuil, Nolwenn Merlet, Teodora Mihalache-Avram, Pascale Geoffroy, Pier-Luc Tardif, Abedelnasser Abulrob, Mehdi Arbabi-Ghahroudi, Feng Ni, Martin Sirois, Philippe L. L’Allier, Éric Rhéaume, Frédéric Lesage, Jean-Claude Tardif

**Affiliations:** 1 Montreal Heart Institute, 5000 Belanger street, Montreal, Quebec H1T 1C8 Canada; 2 Department of medicine, Université de Montréal, 2900 Édouard-Montpetit, Montreal, Quebec H3T 1J4 Canada; 3 Département de Génie Électrique et Institut de Génie Biomédical, École Polytechnique de Montréal, 2900 Édouard-Montpetit, Montreal, Quebec H3T 1J4 Canada; 4 Department of Translational Biosciences, Human Health Therapeutics Research Centre, National Research Council of Canada, 1200 chemin de Montréal, Ottawa, Ontario K1A 0R6 Canada; 5 Department of Downstream Processing and Analytics, Human Health Therapeutics Research Centre, National Research Council of Canada, 6100 Royalmount Avenue, Montreal, Quebec H4P 2R2 Canada

## Abstract

This study aimed to evaluate the feasibility and accuracy of a technique for atherosclerosis imaging using local delivery of relatively small quantities (0.04–0.4 mg/kg) of labeled-specific imaging tracers targeting ICAM-1 and unpolymerized type I collagen or negative controls in 13 rabbits with atheroma induced by balloon injury in the abdominal aorta and a 12-week high-cholesterol diet. Immediately after local infusion, *in vivo* intravascular ultrasonography (IVUS)-NIRF imaging was performed at different time-points over a 40-minute period. The *in vivo* peak NIRF signal was significantly higher in the molecular tracer-injected rabbits than in the control-injected animals (P < 0.05). *Ex vivo* peak NIRF signal was significantly higher in the ICAM-1 probe-injected rabbits than in controls (P = 0.04), but not in the collagen probe-injected group (P = 0.29). NIRF signal discrimination following dual-probe delivery was also shown to be feasible in a single animal and thus offers the possibility of combining several distinct biological imaging agents in future studies. This innovative imaging strategy using *in vivo* local delivery of low concentrations of labeled molecular tracers followed by IVUS-NIRF catheter-based imaging holds potential for detection of vulnerable human coronary artery plaques.

## Introduction

Cardiovascular atherosclerotic events are the leading cause of mortality worldwide^1^ and arise most frequently from coronary plaque disruption, which can trigger acute myocardial infarction and sudden cardiac death^2^. Despite our current knowledge of inflammatory and immune processes in atherogenesis, the early detection of vulnerable plaques and the ability to predict plaque rupture in susceptible patients remain an unmet need in clinical practice^3,4^. Although currently available intravascular imaging modalities enable *in vivo* characterization of morphological parameters of atheroma plaques, they have failed to demonstrate their relevance in preventing acute cardiac events^5–8^.

Near-infrared fluorescence (NIRF) is an innovative high-resolution imaging technology that allows visualization of inflammatory processes at the cellular level using target-specific labeled tracers, thus enabling atherosclerosis detection at early stages of the disease^9–11^. The concept of using specific site-targeted contrast agents for *in vivo* detection of vascular pathology was previously demonstrated in a canine arterial thrombi model using ultrasonic contrast agent^12,13^. In the past decade, NIR-fluorescence catheters have been developed for intravascular imaging that could enable *in vivo* detection of high-risk coronary plaque biomarkers and, consequently, optimize patient stratification and clinical management^14–20^. The current limitations to the clinical translation of NIRF imaging include the lack of clinically approved tracers that target specific features of plaque biology due, in part, to the weak sensitivity to molecular imaging agents, risk of toxicity from systemic administration, costs, labeling and regulatory issues^21^. Indocyanine green (ICG) and methylene blue are near-infrared fluorophore approved by the U.S. Food and Drug Administration (FDA) and have been used for NIRF imaging in both preclinical and clinical studies. Although Verjans *et al*.^22^ demonstrated ICG deposition in carotid plaque specimens from five patients using *ex vivo* intra-arterial dual-modal optical coherence tomography (OCT) – NIRF imaging, results showed less specific binding of the fluorophore to human plaque macrophages and lipids by comparison to previous data published from rabbit atherosclerotic plaques^23^. The discrepancy of ICG specificity observed between rabbits and human atherosclerotic plaque components could result from the short systemic half-life of ICG and the low concentration administered to subjects prior to endarterectomy^22^.

This preliminary study aimed at overcoming the present limitations of NIRF imaging and addressing the need for a clinically available intravascular molecular imaging modality to accurately and safely target both inflammation and specific atherosclerotic plaque components, an unmet need in clinical practice. Accordingly, the feasibility of a technique of local delivery of imaging agents was evaluated using two newly engineered near-infrared labeled molecular probes to image plaque composition and inflammation using a custom bimodal intravascular ultrasound (IVUS) – NIRF imaging catheter system in atherosclerotic rabbit aortas.

## Results

### Colocalization of NIRF signal in atherosclerotic plaques

**Figure 1:**
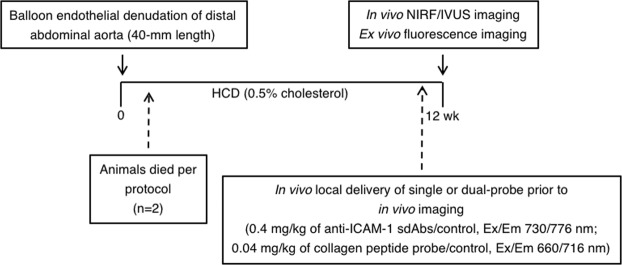
Atherosclerotic rabbit model. At week 0, 15 male NZW rabbits of 12–13 weeks of age underwent balloon denudation of the distal abdominal aorta (40-mm length), upstream of the aorto-iliac bifurcation. At recovery, they were fed with a high-cholesterol diet (HCD) (0.5% cholesterol) over a period of 12 weeks. *In vivo* imaging experiments were performed at week 12, which consisted in performing local delivery of labeled NIRF molecular probes (0.04–0.46 mg/kg/probe) using a porous-balloon catheter at the site of the injured aorta. Immediately after local probe infusion, *in vivo* IVUS-NIRF imaging was acquired and repeated every 10-min for 40 minutes (total of 5 pullbacks/rabbit). Following *in vivo* imaging, *ex vivo* fluorescence imaging and histopathology were performed.

**Figure 2:**
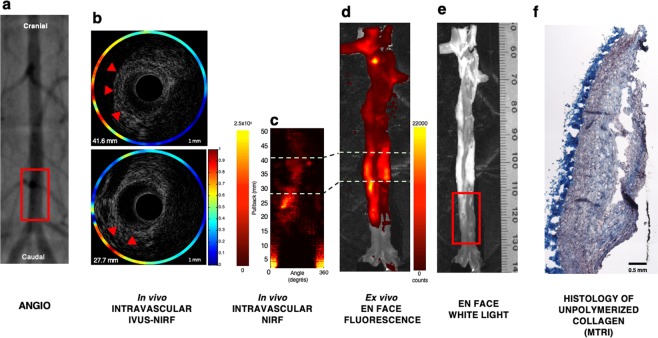
NIRF molecular imaging of unpolymerized type I collagen in atherosclerotic balloon injured rabbit abdominal aortas. Collagen-binding hairpin peptide was injected locally (0.04 mg/kg) in the balloon-injured distal abdominal aorta of atherosclerotic rabbits after 12 weeks of 0.5% cholesterol-enriched diet. (**a**) Shows conventional angiography of the rabbit aorta with the red box indicating the balloon-injured area; (**b**) Represents IVUS imaging cross-sections of the aorta at two distinct pullback distances from the aorto-iliac bifurcation (27.7 mm and 41.6 mm, respectively). Red arrows delineate eccentric atherosclerotic plaques on IVUS imaging, which are colocalized with higher NIRF signal intensity (orange/red, outer color spectrum); (**c**) Shows *in vivo* intravascular NIRF imaging pullback of collagen-binding peptide probe localization in the rabbit abdominal aorta performed 40 minutes after local injection of the NIRF labeled agent; (**d**) Demonstrates *ex vivo en face* fluorescence of collagen-binding peptide over the entire rabbit aorta performed 60 minutes after local injection of the NIRF labeled agent. There are two distinct zones of higher NIR-fluorescence signal intensity seen on either *in vivo* and *ex vivo* NIRF imaging, localized mostly upstream of the injection area (**c**–**e**) Shows the corresponding white light image of the rabbit aorta with the red box indicating the balloon injured area. The distinct zones of higher NIRF signal displayed on both *in vivo* and *ex vivo* images appear to be localized in a lipid-rich plaque area (bright white areas); (**f**) Correlation histopathology of unpolymerized collagen in the distal abdominal aorta following staining with Masson’s trichrome (MTRI). *In vivo* NIRF color scale is in arbitrary units (yellow/white: higher NIRF signal intensity; red: lower NIRF signal intensity). *Ex vivo* NIRF color scale displays number of counts according to signal intensity (yellow/white: higher NIRF signal intensity; red: lower NIRF signal intensity).

Fifteen male New Zealand White (NZW) rabbits underwent balloon denudation in the distal abdominal aorta (40-mm length), followed by a 12-week high-cholesterol diet (0.5% cholesterol). *In vivo* IVUS-NIRF imaging was performed at week 12 following local delivery of labeled NIRF molecular agents targeting ICAM-1 and unpolymerized type I collagen at the injured site of the abdominal aorta (Fig. 1). In order to perform accurate local delivery of targeted molecular probes at the site of the injured abdominal aorta, conventional angiography of the distal aorta was performed to locate the aorto-iliac bifurcation for precise porous-balloon catheter positioning. Figure 2 shows an example of NIRF signal colocalization following local delivery of the collagen-binding peptide probe in the distal abdominal aorta of a diseased rabbit aorta. Figure 2a demonstrates the angiographic location of the balloon injury region (red square) in the distal part of the abdominal aorta, which was the site of local infusion of the molecular imaging agent. Following *in situ* delivery of the imaging tracer, the rabbit aorta was imaged using intravascular IVUS-NIRF imaging that enabled colocalization of *in vivo* NIRF signal with atherosclerotic plaque identified by IVUS (Fig. 2b). We observed that *in vivo* NIRF signal location obtained with the dual-mode imaging catheter matched accurately the signal obtained by *ex vivo* fluorescence reflectance imaging (FRI) imaging of the aorta (Fig. 2c,d). The fluorescence signal was stronger in lipid-rich segments of the aorta, as shown on white light image (Fig. 2e). Staining analysis from sections harboring atheroma plaques revealed that collagen-binding peptide fluorescence colocalized with collagen-rich plaques, as shown on Masson’s trichrome staining (Fig. 2f).

### *Ex vivo* binding of anti-ICAM-1 single-domain antibody (sdAbs) and collagen-binding peptide to antigens in rabbit atherosclerotic plaque

**Figure 3:**
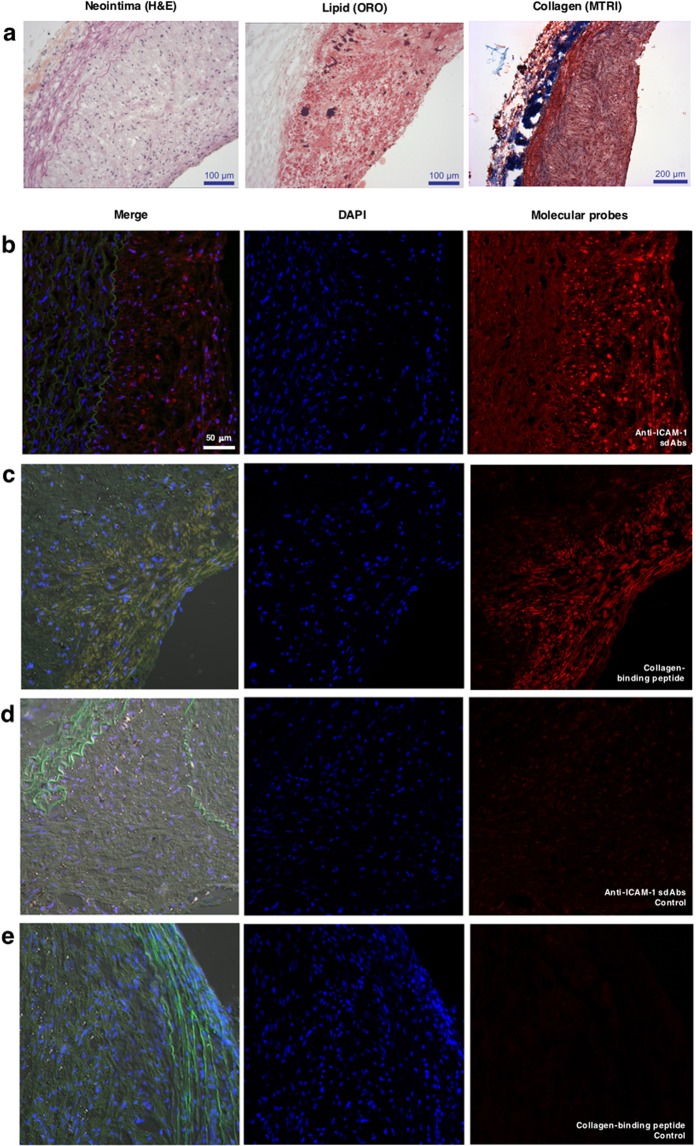
*Ex vivo* binding of NIRF molecular probes targeting ICAM-1 and unpolymerized type I collagen to atherosclerotic lesions in rabbit aortas. Atherosclerotic rabbit model. (**a**) Shows histopathology staining of rabbit denudated aorta sections. Left panel represents neointima staining with hematoxylin and eosin (H&E); mid-panel represents neutral lipids staining with Oil red O (ORO); right panel represents collagen staining with Masson’s trichrome (MTRI). All images are at x4 magnification. Cryosections from atherosclerotic rabbit aorta were stained with: (**b**) anti-ICAM-1 sdAbs; (**c**) collagen-binding peptide; (**d**) anti-ICAM-1 sdAbs control antibody and; (**e**) collagen-binding control peptide. The right panel shows fluorescence signal from either molecular probe targeting ICAM-1 or unpolymerized type I collagen, or the negative control probes. The left panel shows a merged image of both DAPI and the fluorescent molecular probes. The cell nuclei (blue) were stained with DAPI (4′,6-Diamidino-2-Phenylindole,Dilactate).

Balloon denudation of rabbit abdominal aortas, in addition to atherogenic diet, led to the formation of lipid-rich atheroma plaques, as shown on histopathology sections (Fig. 3a). Staining analysis showed the presence of collagen (on Trichrome Masson’s staining) in inflamed plaques, as demonstrated by the presence of Oil Red O (ORO)-positive lipid plaques and neointima (on Hematoxylin and eosin staining). Prior to performing *in vivo* NIRF imaging in experimental atherosclerosis, we confirmed that both NIR-fluorescent molecular probes reacted with antigens in atherosclerotic aorta sections of hypercholesterolemic rabbits. Figure 3b shows focal staining of ICAM-1 localized in the intima of the aorta (merged image), by comparison to very weak signal obtained with the control sdAbs (Fig. 3d). Specific binding of the labeled nanobodies to ICAM-1 at the surface of cellular membranes rather than in the cytoplasm in tumor-necrosis factor alpha (TNF-α) activated human umbilical vein endothelial cells (HUVEC) was previously demonstrated^24^. Diffuse staining of collagen with the collagen-binding peptide was observed in both the intima and media of rabbit atherosclerotic aorta (Fig. 3c) and staining with the control collagen-peptide probe was negative (Fig. 3e).

### Systemic clearance of anti-ICAM-1 sdAbs and collagen-binding peptide following *in vivo* local injection at the site of rabbit injured aorta

**Figure 4:**
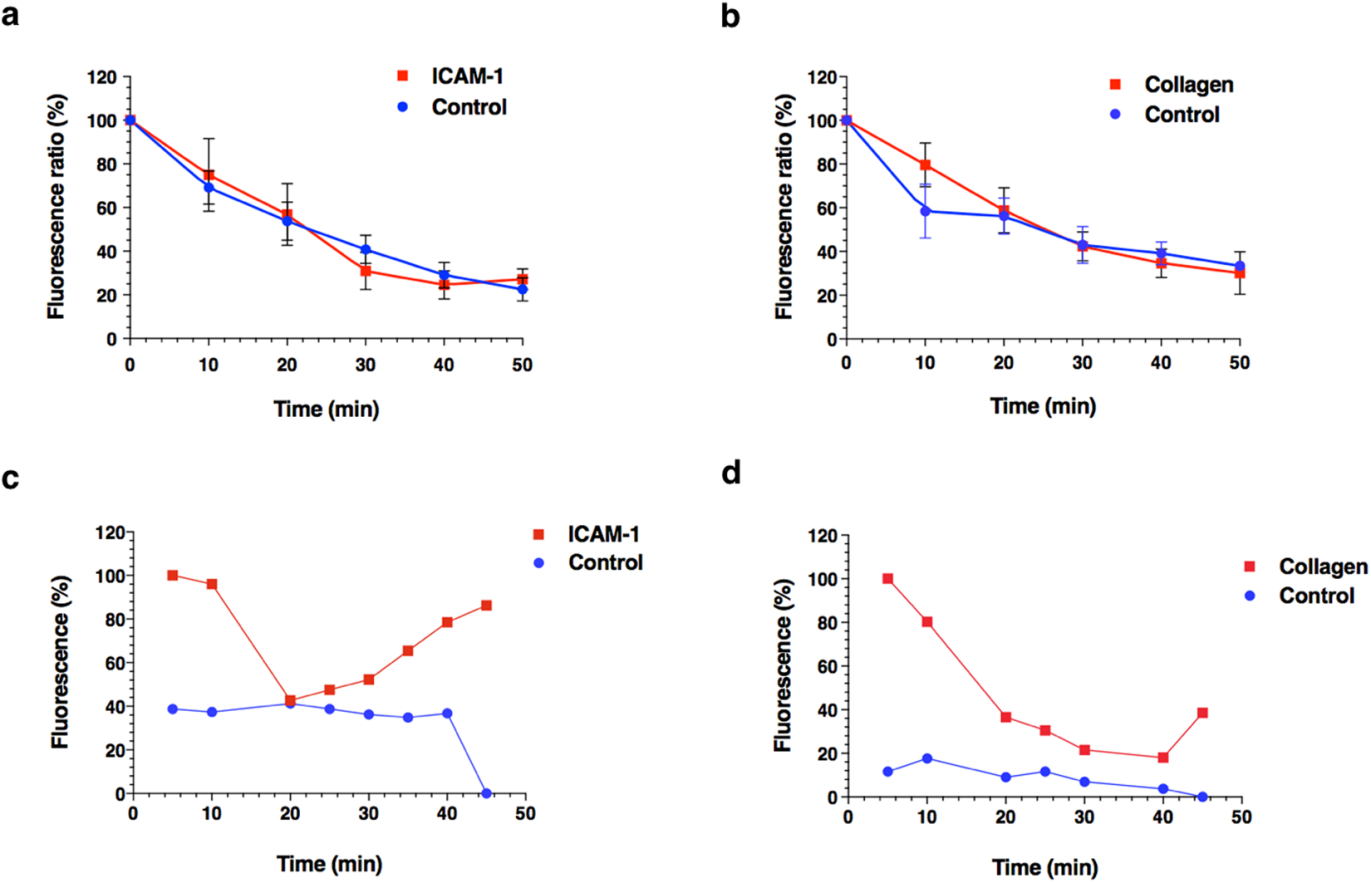
Blood clearance of molecular probes in atherosclerotic rabbits. Clearance from the blood of: (**a**) anti-ICAM-1 sdAbs (red squares) and the corresponding negative control (blue circles) from (n = 5/group) and; (**b**) collagen-binding peptide (red squares) and the corresponding negative control (blue circles) (n = 5 in the collagen group; n = 4 in the control group). Blood clearance kinetics shows a continuous decrease in NIRF signal intensity, which tends to stabilize 40 minutes after *in vivo* local injection of all NIR-fluorescence molecular agents. *In vivo* fluorescence signal detected by IVUS-NIRF catheter of both (**c**) anti-ICAM-1 sdAbs (red squares) and (**d**) collagen-binding peptide, by comparison to their negative controls (blue circles). Both imaging probes shows an initial strong NIRF signal that decreases rapidly, followed by a re-increase in signal intensity between 20–40 minutes, mostly seen with anti-ICAM-1 sdAbs. By contrast, a weak fluorescence signal is observed for both negative controls following *in vivo* local injection in the injured zone, which then becomes undetectable after 45 minutes. Data points are mean ± standard error of the mean (SEM) from the rabbits combined.

We assessed blood clearance of both molecular agents and their controls following *in situ* injection at the balloon-induced injury area of rabbit abdominal aorta. Serial heparinized blood samples were taken from the sheath catheter immediately following local delivery of the NIRF probes using a porous balloon and every 10 minutes to perform *ex vivo* fluorescence imaging quantification. The blood clearance kinetics of both anti-ICAM-1 sdAbs (Fig. 4a) and collagen-binding peptide probes (Fig. 4b) demonstrate a rapid systemic decline during the initial 30 minutes, which begins to stabilize at 40 minutes. There was no difference in blood kinetics between the molecular agents and their respective controls. Moreover, simultaneous dual-probe delivery did not alter the systemic clearance of the imaging agents by comparison to single-probe delivery (data not shown). Following local delivery of the imaging tracers, the first pullback performed using the IVUS-NIRF catheter detected a strong saturated NIRF signal *in vivo* that rapidly declined in the first 20 minutes for both probes, followed by a re-increase in signal intensity between 20 to 40 minutes, by comparison to a continuous slow decline of signal intensity of the control probes (Fig. 4c,d). The absence of binding of the control tracers to the arterial wall resulted in a continuous drop in signal intensity at every time-point that was then undetectable at 45 minutes. The specific binding of molecular tracers to atheroma plaques generated a strong NIRF signal of variable intensity that could be accurately imaged by the intravascular imaging catheter after 40 minutes.

### Comparison between *in vivo* and *ex vivo* near-infrared fluorescence (NIRF) imaging of rabbit atheroma plaques using both anti-ICAM-1 sdAbs and collagen-binding peptide probes

**Figure 5:**
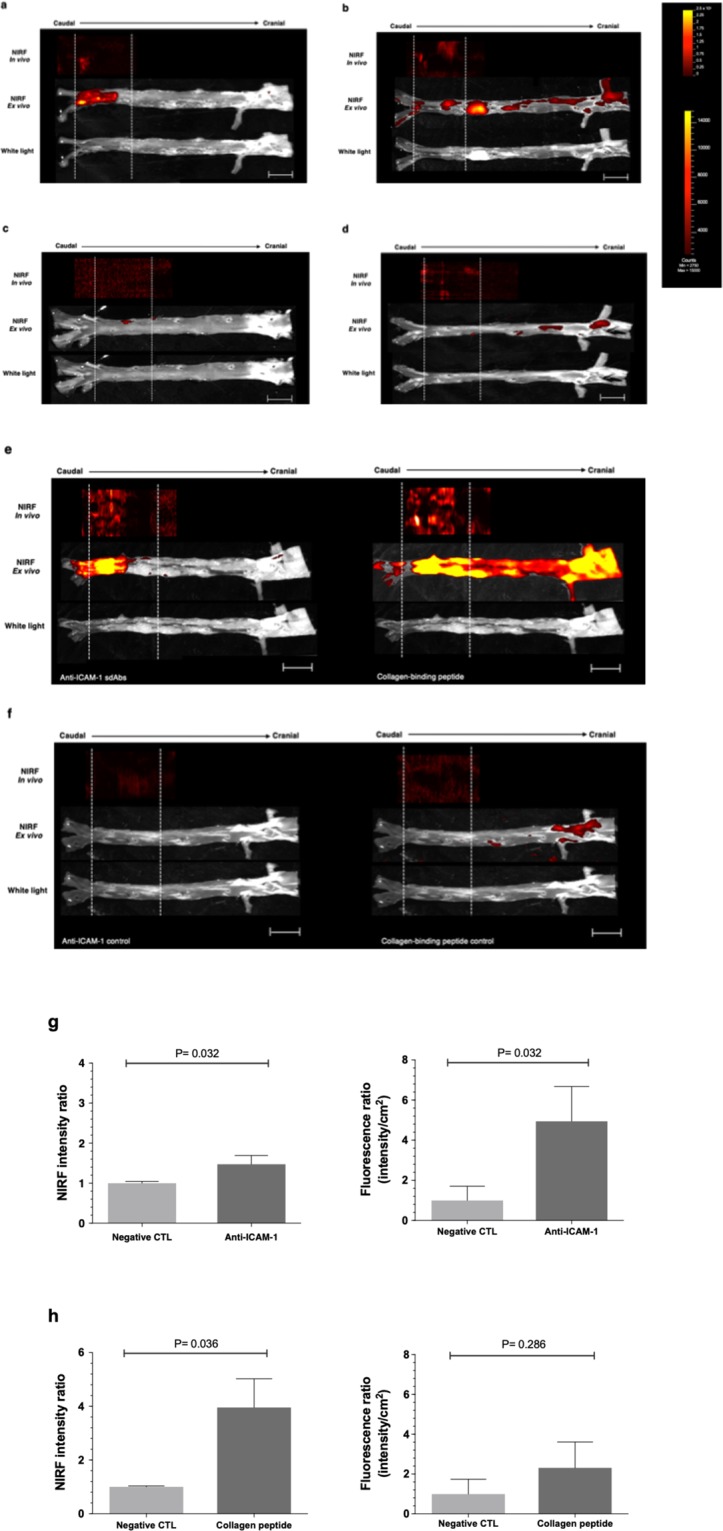
Comparison of *in vivo* and *ex vivo* near-infrared fluorescence (NIRF) imaging of targeted ICAM-1 and collagen in atherosclerotic rabbit aortas. Comparison of *in vivo* NIRF imaging and *ex vivo* fluorescence signal following single-probe local injection of either: (**a**) anti-ICAM-1 sdAbs, (**b**) collagen-binding peptide, (**c**) ICAM-1 negative controls; (**d**) collagen negative control; (**e**) dual-probe local injection of anti-ICAM-1 sdAbs combined to collagen-binding peptide probe, or (**f**) the combined negative controls. *In vivo* and *ex vivo* signals were colocalized in both single- and dual-probe injections along the longitudinal direction and delineated by white dot lines. *In vivo* and *ex vivo* fluorescence signals were properly aligned, taking into account post-mortem average tissue shrinkage estimated between 25–30%. In both groups, NIRF signal was only detected over the balloon-injured zone with the anti-ICAM-1 sdAbs agent (**a**,**e**). However, fluorescence signal was observed upstream from the injection zone with the collagen-binding peptide probe, notably in an aneurysm lipid-rich area of the aorta, as shown in (**b**,**e**). There were no NIRF signals detected in the control-injected groups (**c**,**d**,**f**), except in the abdominal and thoracic aorta of 2 rabbits following dual-injection of the control collagen-specific agent. *In vivo* and *ex vivo* peak NIRF signal in: (**g**) ICAM-1 probe- and (**h**) collagen-peptide probe injected abdominal aortas. White dot lines indicate the injury zone of the distal abdominal aorta, site of molecular probes delivery through the porous-balloon catheter. *In vivo* NIRF color scale is in arbitrary units (yellow/white: higher NIRF signal intensity; red: lower NIRF signal intensity). *Ex vivo* NIRF color scale displays number of counts according to signal intensity (yellow/white: higher NIRF signal intensity; red: lower NIRF signal intensity). Scale bar: 10 mm. Data is presented as mean ± standard error of the mean (SEM).

Intravascular imaging was safely performed in all 13 atheromatous rabbits. As both ICAM-1 and unpolymerized type I collagen specific probes bind to rabbit atherosclerotic plaques, we evaluated the feasibility of local injections of these tracers to locate plaque in diseased animals as a potential novel modality for accurate and safe catheter-based NIRF detection. In order to determine the optimal timing for NIRF imaging, dual-modal intracoronary IVUS-NIRF imaging was performed serially following local delivery of molecular probes over a 40-minute period. Based on *in vivo* fluorescence signal stability, IVUS-NIRF imaging analysis acquired at 40 minutes revealed focal NIRF signals within the injured zone following either single (Fig. 5a–d) or dual-tracer infusion (Fig. 5e,f). Specific probe-labeling using IR800CW and Alexa-660, distinct fluorophores with different emission wavelength properties, enabled dual-probe injection and *in vivo* and *ex vivo* NIRF signal differentiation in a single diseased animal. Immediately after *in vivo* IVUS-NIRF imaging, fluorescence reflectance imaging of resected aortas was performed and revealed NIRF activity predominantly in lipid-rich regions visible in white light images of atheroma-bearing aortas, whereby signal intensity tended to correlate with plaque burden. Interestingly, focal and diffuse NIRF signals from non-injured lipid-rich arterial segments were observed in the collagen probe-injected atheroma rabbits. Concordance between *in vivo* and *ex vivo* NIRF signal location was evaluated by comparing NIR-fluorescence signal on intravascular imaging pullbacks and resected aortas using anatomic landmarks, whereas results are displayed with a shift between fluorescence signals due to postmortem tissue shrinkage (average of 25–30%). *In vivo* and *ex vivo* NIRF signals accurately matched in all rabbits for both labeled-specific tracers (Fig. 5a,b,e). By contrast, there were no NIRF signals detected in the control-injected groups (Fig. 5c,d,f), except in 2 collagen probe-injected diseased animals that showed NIRF signal in high-density plaque regions upstream from the injection site (thoracic and abdominal aorta). For the ICAM-1 targeted probe, the *in vivo* peak NIRF signal was 1,47-fold greater than the control (1.47 ± 0.22 versus 1.00 ± 0.05; *P* = 0.032; Fig. 5g) and *ex vivo* peak NIRF signal was 4.9-fold greater than the control (4.94 ± 1.73 versus 1.00 ± 0.71; P = 0.032; Fig. 5g). By contrast, *in vivo* peak NIRF signal for the unpolymerized type I collagen targeted peptide probe was 3.9-fold greater than the control (3.96 ± 1.07 versus 1.00 ± 0.03; P = 0.036; Fig. 5h) and *ex vivo* peak NIRF signal was 2.3-fold greater than the control but not statistically significant (2.31 ± 1.30 versus 1.00 ± 0.74; P = 0.286; Fig. 5h).

### Colocalization of microscopic NIRF signal of ICAM-1 with endothelial cells localized in rabbit atheroma plaques

**Figure 6:**
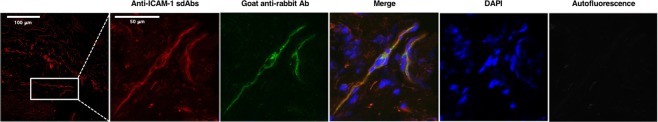
Immunostaining validation of anti-ICAM-1 sdAbs in atherosclerotic rabbit aortas. Colocalization of ICAM-1 in atherosclerotic rabbit aorta by immunofluorescence. Anti-ICAM-1 sdAbs NIRF signal colocalize with ICAM-1 on endothelial cells by staining the sections with primary mouse anti-rabbit ICAM-1 monoclonal antibody (red NIRF signal, images 1 and 2), followed by a secondary labeling using goat anti-mouse IgG1 antibody (green NIRF signal, image 3), The merged image shows specific colocalization of ICAM-1 on endothelial cell surface of atherosclerotic aorta segments (yellow NIRF signal, image 4). The endothelial cell nuclei (blue) were stained with DAPI (image 5). No autofluorescence signal was detected under confocal microscopy (image 6).

Immunostaining analysis was performed to further validate colocalization of NIRF signal of ICAM-1 on endothelial cells in atheroma plaques following local infusion of anti-ICAM-1 sdAbs imaging tracer. Fig. 6 shows strong NIRF signal with confocal microscopy in the balloon-induced injured area of aortas following anti-ICAM-1 sdAbs colocalization with mouse anti-rabbit ICAM-1 monoclonal antibody (red NIRF signal), and subsequent incubation with goat anti-mouse IgG1 cross-absorbed secondary antibody (merge image, yellow NIRF signal).

## Discussion

In the present study, we demonstrated the feasibility of local delivery of relatively small quantities of NIRF targeted tracers for *in vivo* imaging of both morphological and biological biomarkers of atherosclerotic plaques using a fully-integrated real-time intravascular bimodal IVUS-NIRF imaging catheter. This preliminary study describes an application using *in vivo* molecular agents for invasive imaging of both inflammation and atheroma plaque components that could be translatable for future detection of high-risk human coronary artery plaques.

*In vivo* NIRF catheter signal detection was obtained following *in situ* delivery of molecular agents targeting either ICAM-1 or unpolymerized type I collagen in the balloon-induced injured area of the distal abdominal aorta of rabbits, as well as upstream of the injected region with the collagen-binding peptide agent. *In vivo* NIRF signals colocalized with atherosclerotic plaques visualized by IVUS, and signal intensity correlated with plaque burden. The use of a microporous-balloon catheter for *in situ* fluorescent probe injection, as well as repeated *in vivo* IVUS-NIRF imaging pullbacks in a single animal, demonstrated the feasibility and safety of the technique and enabled determination of NIRF molecular imaging probe kinetics for optimal *in vivo* catheter-based imaging. The use of a low-pressure microporous balloon-catheter system confers the advantage of both bringing imaging probes in contact with the intima, while avoiding plaque disruption or vessel trauma from its semi-compliant properties that eluded balloon over-expansion. Moreover, performing proper balloon sizing to vessel diameter by QCA prior to local infusion renders this technique suitable for translational applications in future studies. The absence of *in vivo* NIRF signal at the vicinity of atheroma plaques following injection of specific negative controls further validated the specificity of fluorescence signal obtained with both ICAM-1 and unpolymerized type I collagen targeted imaging tracers. These findings were then confirmed by matching the whole rabbit aorta fluorescence reflectance imaging *ex vivo* with *in vivo* NIRF signal detected by intravascular NIRF, which showed signal colocalization in all rabbits with respect to both molecular imaging agents.

*In vivo* imaging tracer kinetics were analysed following multiple pullbacks performed at different time points using a custom dual-imaging catheter. Differences observed between *in vivo* clearance kinetics of molecular tracers and their controls could be the result of both tracer’s initial local diffusion of bounded probes on plaque surface, along with systemic clearance of unbound probes, leading to an initial decrease in signal intensity. A subsequent increase in fluorescence signal observed with both target-specific tracers in the aftermath of additional binding of the probes from systemic circulation/redistribution was seen, more predominantly with anti-ICAM-1 sdAbs, reaching a state of stability between 40 to 50 minutes. It is plausible that the peptide collagen probe diffused more easily in the plaque than ICAM-1 nanobodies immediately following local delivery and thus a faster saturation of the ligands was observed. Moreover, from the *ex vivo* imaging of the whole rabbit aortas, we’ve observed focal and diffuse NIRF signal from non-injured lipid-rich arterial segments, mostly in the thoracic aorta, which may explain less redistribution of the peptide probe at sites of the injured lipid plaques in subsequent imaging time points. Nevertheless, pharmaco-kinetic properties of both imaging tracers enable targeting diseased arterial segments prior to achieving a steady state, which makes them valuable for future studies in atherosclerosis imaging.

Numerous fluorophore-coupled imaging probes targeting a variety of molecular biomarkers have been previously tested in atherosclerotic animal models for intravascular imaging, including matrix metalloproteinases^25,26^, macrophages^23,26^, cathepsins^14–16,26–28^ and oxidized low-density lipoproteins (oxLDL)^29^. Systemic administration of a prespecified dosage of these imaging compounds several hours prior to *in vivo* and/or *ex vivo* imaging was performed to allow fluorescent probe diffusion in the atheroma plaques. Indocyanine green was also used as a fluorescent probe in experimental animal models (10 mg/kg in rabbits) and humans (0.25–2 mg/kg) for the assessment of high-risk plaques following intravenous injection of safe doses^17,18,22,23^. By contrast, our study demonstrated the feasibility and reliability of specific *in vivo* NIRF signals following local delivery of a concentration 6–50 times less than the intravenous dosage of molecular fluorescent tracers (0.04–0.4 mg/kg), with the advantages of avoiding potential risk of adverse effects or immune reactions from systemic administration, suboptimal biodistribution and tracer diffusion in regions of interest. As shown in atherosclerotic rabbits following local injection of the collagen-peptide probe, *in vivo* NIRF signals were observed not only in the balloon-induced injury area but also in several lipid-rich regions of the aorta. This suggests that the use of another vector for selective *in vivo* probe delivery for future studies in coronary arteries could enable accurate imaging of biological biomarkers of surrounding atheroma plaques while avoiding the relative risk of vascular injury or compromising blood flow from *in situ* tracer delivery. The injection through a 6 F guiding catheter could be a plausible alternative however would enable a semi-selective injection of tracers rather that a local selective infusion in the region of interest. Perhaps the use of a microcatheter or thrombectomy catheter available in the catheterization laboratory would convey the injection of imaging probes at the vicinity of the plaque, thus render a more selective injection without the risk of ischemia or plaque disruption. From a translational standpoint, this technical application could certainly outweigh the limitations of conventional angiography in ascertaining the true extent of disease in the arterial wall, and for better assessment of non-flow limiting coronary stenoses harboring features of plaque instability.

A postmortem study revealed that plaque rupture was by far the most frequent cause of atherothrombosis, accounting for two-thirds of sudden cardiac deaths and more than 65% of acute myocardial infarctions^30^. Rupture-prone plaques are defined by a large lipid-rich necrotic core with an overlying thin fibrous cap infiltrated with macrophages and lymphocytes. The vulnerable plaque, or so-called thin-cap fibroatheroma (TCFA), is subjected to factors that can alter plaque morphology and thus lead to plaque instability and rupture^30,31^. Although the PROSPECT (Providing Regional Observations to Study Predictors of Events in the Coronary Tree) study identified specific morphologic predictors of lesion progression in non-culprit plaques at 3 years assessed by virtual histology (VH-IVUS)^5^, findings that were further supported by the VIVA (VH-IVUS in Vulnerable Atherosclerosis) and ATHEROREMO-IVUS (European Collaborative Project of Inflammation and Vascular Wall Remodeling in Atherosclerosis-Intravascular ultrasound) studies^6,8^, plaque morphology and composition alone do not accurately predict which non-culprit plaques will cause recurrent adverse cardiac events. Thus, routine use of invasive imaging to screen for vulnerable plaques and to predict the risk of cardiovascular complications is currently proscribed^32^. This reinforces the potential of imaging active biological processes to provide new insights on atheroma plaque growth, healing and biomarkers involved in plaque instability. In the present study, ICAM-1 and unpolymerized type I collagen were the selected biomarkers for *in vivo* molecular imaging of both inflammation and plaque morphology. With the knowledge that ICAM-1 is involved in plaque formation and progression, while collagen, a major constituent of atheroma plaque, undergoes proteolysis and is less produced by smooth muscle cells (SMC) in high-risk plaques, the quantification of both biomarkers by IVUS-NIRF imaging could enable characterization of plaque morphology and harbor the potential to discriminate between stable and unstable plaques at risk of rupture, along with other inflammatory biomarkers. We have demonstrated that dual injection of molecular probes coupled with tracers harboring different excitation and emission wavelength properties enabled accurate NIRF signal discrepancy in a single animal. Perhaps the combination of several biological fluorescent agents, along with plaque morphology assessed by catheter-based technology (IVUS or OCT), may be an attractive avenue for identifying *in vivo* high-risk plaques in future studies.

The current study describes a reliable technique for imaging specific features of atherosclerotic plaques using local delivery of labeled specific molecular tracers followed by intravascular IVUS-NIRF imaging. Although this proof-of-principle study using novel molecular imaging probes is promising for identifying both structural and biological properties of atherosclerotic plaques, these preliminary findings should be validated in a longitudinal study using a larger animal model that features coronary atheroma with a phenotype more similar to that observed in humans (e.g. swine). Such studies may provide new insights on plaque inflammation at early stages of the disease. Given that both ICAM-1 and unpolymerized type I collagen have not yet been established as being indicative of plaque rupture and thrombotic complications, the application of this imaging methodology using a combination of newly designed tracers targeting these biomarkers could pave the way for a better understanding of the pathophysiology of high-risk atheroma plaques and disease progression, especially the contributions of plaque inflammation and tissue (collagen) remodeling, and may allow to develop future targeted therapies (personalized medicine).

## Limitations

Notwithstanding that this study demonstrates the feasibility of *in vivo* labeled tracer-based IVUS-NIRF intravascular molecular imaging as a method for detection of inflamed atherosclerotic plaques, there are several limitations to be mentioned. First, we were unable to conduct correlation analysis of quantified NIRF signal between *in vivo* and *ex vivo* fluorescence signal detected in rabbit aortas due to the small sample size. Second, despite *in vivo* and *ex vivo* blood clearance kinetics analyses of both molecular agents for optimal timing of *in vivo* catheter-based imaging, their diffusion properties from plaque surface remain unknown. Since a single concentration of all tracers was tested in the present study, it remains to be determined whether a lower dose using the same administration method would provide sufficient NIRF enhancement for *in vivo* catheter-based signal detection. In addition, *in vivo* imaging was performed at different time-points over a 40-minute period and, from the late resurgence in signal intensity detected by the imaging catheter *in vivo* for both probes, the optimal timing for intravascular imaging may be beyond this timeline. I*n vivo* NIRF signal was obtained with 2 collagen negative control probes in lipid-rich regions of the aorta, which probably was a result of nonspecific binding of the tracer to plaque surface which may bear an endothelial surface more permeable than non-diseased regions. Another limitation is the large diameter of the current bimodal IVUS-NIRF imaging catheter prototype due to the rigid optic fiber, which limited catheter-to-wall distance in rather small diameter distal abdominal aortas. Nevertheless, further optimization of catheter dimensions and flexibility, in addition to the validation of this novel imaging technique in a porcine atherosclerotic model with plaque biology more similar to human fibroatheroma plaques, will be required to overcome these limitations.

## Methods

### Animal model and experimental protocol

The atherosclerotic plaque model was generated by performing balloon denudation in the distal abdominal aorta of 15 male New Zealand White rabbits (3 kg, aged 12–13 weeks; Charles River Laboratories, Saint-Constant, QC, Canada) at week 0. Rabbits were then placed on a cholesterol-enriched diet (0.5% cholesterol, Harlan Teklad Diets, Madison, WI) for 12 consecutive weeks (Fig. 1). The experimental protocol was approved by the animal ethics committee of the Montreal Heart Institute Research Center according to the guidelines of the Canadian Council on Animal Care.

### Balloon denudation

Under continuous anesthesia (aceprozamine (1 mg/kg intramuscular (IM)), buprenorphine (0.01 mg/kg IM) and inhaled isoflurane (4% v/v, Baxter, Deerfield, IL)), a 5 French (F) sheath catheter (Cordis Corporation, Miami, FL, USAs) was used to cannulate the right carotid artery. Baseline angiography of the abdominal aorta (Siemens, Germany) was performed using a 5 F right Judkins (JR) 4.0 guiding catheter (Cordis Corporation, Miami, FL, USA) inserted into the aorta over a 0.014-inch guidewire (Abbott Vascular, Santa Clara, CA). The minimal lumen diameter (MLD) of the abdominal aorta, measured on an end-diastolic frame by quantitative coronary analysis (QCA; Syngo software, ARTIS Siemens, Germany), was used for accurate PTCA balloon sizing. Under fluoroscopic guidance, balloon injury was performed in the distal 40-mm of the abdominal aorta using a standard PTCA balloon-catheter (Boston Scientific, Marlborough, MA), corresponding to a balloon/artery ratio of 1.1 to 1.2:1, that was inflated at 10 atm for 30 seconds over three consecutive times, with 30-second intervals between inflations. After balloon denudation, the sheath catheter was removed, and the right carotid artery was ligated. Post-operative care consisted in strict monitoring of the vital signs and animal behavior. Analgesia was managed by subcutaneous administration of buprenorphine (0.02 mg/kg) and ketoprofen (2 mg/kg) immediately after the procedure and on day 1 (2 doses). Post-operative infection management consisted in a prophylactic dose of Borgal (67 mg/kg) administered subcutaneously immediately after the surgery combined to a sterile dressing applied on the surgical wound, with daily surveillance of the surgical sutures.

### Anti-ICAM-1 single-domain antibody

Anti-ICAM-1 V_H_H single-domain antibody (sdAb) or nanobodies were generated by immunizing a male lama. A V_H_H library was constructed and panned as described before^29^. After four rounds of panning against recombinant human ICAM1 (R&D systems), ICAM-specific V_H_H were identified by phage-ELISA. Unique V_H_H sequences, identified by sequencing, were sub-cloned into pSJF2 expression vector and the V_H_Hs were expressed in TG1 bacteria as described before (US patent: 8,623,369 B2)^29^. The ICAM-1 sdAbs were purified by IMAC column and size-exclusion chromatography (SEC), and their binding affinities were determined by surface plasmon resonance (SPR)^29^. The SEC-purified sdAbs were labeled with IRDye^®^ 800CW (LI-COR Biosciences, MA, USA) infrared dye for NIRF imaging. An ELISA was used to confirm functional reactivity of ICAM-1 sdAb before and after labeling. Briefly, wells of a Nunc96 plate were coated with the recombinant human ICAM and blocked with Strating Block (Thermo Scientific). Serial dilution of sdAbs were added to the wells and incubated for 1 hour at room temperature. Specific bindings of sdAbs were detected by rabbit anti-His tags-HRP conjugate (Bethyl lab), the color was developed by adding KPL substrate (KPLGaithersburg, MD) and the plate was read at 450 nm^33^. As controls, wells with no antigen (blank) and coated by ICAM-1 but used a nonrelated V_H_H were included in the assay.

### Collagen-binding linear hairpin (CBLH) peptide

This probe was derived from a collagen-binding hairpin peptide GEWTWDDATKTWTWTE that targets specifically unpolymerized type I collagen (US patent publication: 2016/0168210 A1). The peptide portion of this probe GEWTWDDATKTWTWTEGGC was purified using HPLC from materials obtained by solid-phase peptide synthesis (SPPS) and labeled via the Cys residue with Alexa-660-monomaleimide (Thermo Fisher Scientific, MA, USA) infrared dye for NIRF imaging. A negative control peptide ETVTFTKTADDYTSEGGGC was derived from a non-binding peptide GEWTYDDATKTFTVTE (US patent publication: 2016/0168210 A1) with the Trp (W) residue replaced by Ser followed by sequence reversal. The control peptide was also conjugated to Alexa-660-monomaleimide. Both probes were further purified using HPLC after Alexa-660 conjugation and dried to powders before reconstitution in physiological saline. Final concentrations of the peptide probes were determined by use of the dry weight of the peptide powders.

### Rabbit atherosclerotic aorta sections

Cryosections from rabbit atherosclerotic aortas were used to test the reactivity of both the anti-ICAM-1 sdAb and unpolymerized type I collagen peptide probes, with their respective negative controls, by *ex vivo* fluorescence microscopy. Cryostat sections of 8 μm were obtained and incubated with 10 μg/ml of either probe for 2 hours at room temperature. Images were taken at 20x with a confocal microscope Zeiss LSM 7 Duo (Carl Zeiss Microscopy GmbH, Göttingen, Germany) with filter sets for Alexa-660 and IR800CW fluorescence signal detection (excitation/emission, 633/641–759 nm).

### *In vivo* intravascular ultrasound (IVUS)-near-infrared fluorescence (NIRF) imaging catheter system

The 5 F compatible custom-made imaging catheter used in this study has been previously described^34,35^. The catheter combines a 400 μm optical fiber that enables 360-degree fluorescence imaging and photoacoustic excitation, and an ultrasound transducer (extracted from the commercially available IVUS 45 MHz Revolution catheter; Volcano Therapeutics, Rancho Cordova, CA) for acoustic imaging and photoacoustic signal detection. The catheter has a diameter of 1.4 mm and is connected to an electronic circuit that enables synchronized acquisitions of fluorescence and IVUS imaging modalities from rotating pullbacks at an angular speed of 30 revolutions/second. The pullback speed was set to 0.5 mm/second and the frame rate was 10 images/seconds, with a total pullback length of 100 mm. Raw data is transferred to a computer bearing a custom Matlab (The MathWorks, Inc., Natick, MA) user interface that filters the signals, reconstructs, and displays the images in real time during acquisition. This single IVUS-NIRF system enable to interchange lasers and filters according to the fluorophore properties to be imaged. Fluorescence excitation was performed using either a 730-nm laser diode for IR800CW labeled probe or a 660-nm laser diode for Alexa-660 labeled probe. We’ve measured Alexa-660 with the 800-nm channel and IR800CW with the 660-nm channel with the imaging catheter and no bleed-through was detected. Emission was detected by a photomultiplier tube (Hamamatsu Photonics, Hamamatsu City, Japan) combined to either a 776-nm longpass filter (IR800CW) or a 716 ± 20 nm filter (Alexa-660).

### *In vivo* near-infrared fluorescence (NIRF) imaging procedure

Of the 15 animals, two died after balloon denudation and were excluded; 13 animals were used to perform *in vivo* imaging procedures at week 12. Under general anesthesia, a 5 F sheath catheter (Cordis Corporation, Miami, FL, USA) was used to cannulate the left carotid artery. Under fluoroscopic guidance, molecular probes were injected at the injured site of the distal abdominal aorta upstream from the aorto-iliac bifurcation using a 20-mm-long semi-compliant microporous balloon catheter (ClearWay^TM^ RX, Atrium Medical, New Hampshire, USA)^36^ inserted over a 0.014-inch guidewire (Abbott Vascular, Santa Clara, CA). Administration dosage for the collagen-targeting probe was 0.07–0.12 mg per rabbit (formulated in an infusion volume of 2.5 ml), which was set according to the probe dosage of 0.25–0.5 mg/kg/probe in rat or mouse systemic biodistribution studies (US patent publication: 2016/0168210 A1). The anti-ICAM-1 sdAb dosage was 0.4 mg/kg per rabbit. Accurate porous-balloon catheter sizing (balloon/artery ratio of 1.1 to 1.2:1) was determined by the minimal lumen diameter (MLD) of the abdominal aorta measured on an end-diastolic frame by quantitative coronary analysis (QCA; Syngo software, ARTIS Siemens, Germany). The infusion was done over 90-sec, at 4 atm, followed by a flush of saline (1.5 mL). Seven rabbits had a single injection of either the probe targeting ICAM-1, unpolymerized type I collagen, or their respective negative controls at the injury site of the distal abdominal aorta; six rabbits had a dual-probe injection of either the combined molecular tracers or the combined controls. Immediately after the injection, the intravascular imaging catheter was inserted over a 0.014-inch guidewire and *in vivo* IVUS-NIRF imaging of the distal abdominal aorta was acquired every 10 minutes over a 40-minute period. For dual-probe injection, 2 consecutive pullbacks were performed at every time-points with different bandpass optical filters for fluorescence detection of both tracers. A total of 5 serial pullbacks were performed in the same arterial segment for each rabbit, whereas the aorto-iliac bifurcation corresponds to the proximal anatomic landmark of the 50-mm length pullback. Blood samples were taken from the sheath catheter (1 mL) prior to each pullback to perform *ex vivo* fluorescence imaging. At the end of the experiment, animals were sacrificed and the aorta was resected.

### *In vivo* intravascular ultrasound (IVUS)-near-infrared fluorescence (NIRF) image analysis

A dedicated blood attenuation algorithm was applied to all *in vivo* pullbacks, as previously described^35^, to compensate the fluorescence signal amplitude with the distance between the imaging catheter and the arterial wall.

### *Ex vivo* fluorescence imaging (FRI) and histopathology

After euthanasia and saline infusion, aortas were resected, opened and manually elongated in a petri dish coated with black wax and filled with saline to perform *en face* FRI at 37 °C using a commercial epi-illumination fluorescence imaging system (IVIS Lumina II, PerkinElmer, MA, USA). The following parameters were used: Ex/Em 745 ± 20/800 nm ± 20 nm for anti-ICAM-1 sdAbs and 640 ± 20/720 ± 20 nm for collagen-binding peptide probe, a field of view (FOV) of 10 cm, F/stop 1, Binning (M) 4/2, excitation time of 20 seconds. The total fluorescence values (intensity/cm^2^) of the region of interest (i.e. 20-mm injected area in the distal abdominal aorta) were analysed using a computer-based digitizing image system software (Lumina II Living Image 2.0). After each imaging session, the regions of interest (ROI; 15 mm in length) were embedded in Neg-50 (Thermo Fisher Scientific, MA, USA) and preserved at −80 °C for further microscopy validation of the *ex vivo* imaging results. The average tissue shrinkage was estimated between 25–30%, according to side branches that were used as landmarks and pullback length. For histopathology analyses, several 6-μm sections were cut, and morphometry was demonstrated with Masson’s trichrome staining, including collagen and fibrosis quantification. Von Kossa staining was performed to identify calcium in the neointima and media, and Oil red O (ORO, Sigma) to identify neutral lipids in the neointima and media.

### Immunohistochemical staining

Cryosections (10 μm thickness) were obtained from fresh-frozen rabbit aortas. Fluorescence microscopy of aorta sections comprised in the region of interest was performed using a Zeiss LSM 7 Duo microscope (Carl Zeiss Microscopy GmbH, Göttingen, Germany) that visualized the distribution of anti-ICAM-1 NIRF signal (Ex/Em, 638/755 nm) and anti-ICAM-1 antibody (Ex/Em, 544/649 nm). NIRF signal colocalization was performed using a mouse anti-rabbit ICAM-1 monoclonal antibody, clone Rb2/3 (kindly provided by Pr. Miron Cybulsky, University of Toronto, Canada). Slides were fixed for 5 minutes in acetone at −20 °C and incubated overnight with primary antibody at 4 °C, followed by staining with a goat anti-mouse IgG1 cross-absorbed secondary antibody, Alexa Fluor 555 (A-21127, Invitrogen, Thermo Fisher Scientific, MA, USA).

### Statistical analysis

Data are presented as mean ± SEM (standard error of the mean). Mann-Whitney test was used to compare peak plaque TBR between the molecular probe injection group and the control injection group for both *in vivo* and *ex vivo* fluorescence studies using Prism 7.0 (GraphPad Software, Sand Diego, CA). Values of *P* < 0.05 were considered statistically significant.

## Data Availability

All data generated or analyzed during this study are included in this published article.
